# 

**Published:** 1874-07

**Authors:** 


☞ Write your Names, Post Office, County and State *plainly*. Be sure to say what Post Office you wish the RECORD sent, and always date your letters. We receive many well written letters, but doubt even the authors being able to decipher their signatures.

☞ Address all Communications, to POWELL & GOLDSMITH, none other.

☞ Send Money by Check, Postal Order or by Registered Letters. We are willing to endorse for the honesty of our subscribers, but cannot be responsible for the irregularity of the mails.

☞ We take pride in the manner in which the SOUTHERN MEDICAL RECORD is put up for mailing. Each number is promptly transmitted to our subscribers by mail, securely wrapped, and plainly addressed.

Our patrons will know therefore, that in case any number should be missing it is not our fault. To accommodate our friends we will endeavor to duplicate any number of the RECORD which may accidentally be *lost* in transmission. P.

☞ Owing to a pressure of outside duties, professionally and otherwise, we have recently been a little behind in our correspondence with patrons, and have neglected to perform requests that have been made of us, as promptly as we should like to have done.

We believe, however, that we are now abreast of our duty in this respect, but should any one of our numerous correspondents have been overlooked, we sincerely ask their pardon for the unintentional slight and hope that they will at once renew whatever request they may have made.

We shall at all times be ready to comply, and will cheerfully answer communications or endeavor to fulfill the wishes of our friends and correspondents. Our work is one of love. The only reward we ask or desire is appreciation, sympathy and coöperation.

P.

*The Examining Board to Regulate the Licensing of Physicians and Druggists in Georgia.*—This Board, which has been in existence since 1824, and whose annual meetings take place at Milledgeville, held a call-meeting of four days in Atlanta, beginning July 14th. This was done for the accommodation of Physicians and Druggists in this and surrounding portion of the State. Twenty-eight or thirty applicants presented themselves, and twenty-three or four were licensed as Druggists and Apothecaries, and one only as Pharmaceutist.

The Board is constituted of a number of eminent medical gentlemen, representing different parts of the State.

The following names composed the Board, present at this call-meeting: Dr. S. G. White, the President, Dr. G. D. Case, Mr. J· M. Clark, Pharmaceutist, all of Milledgeville; also, the senior Editor of this Journal. All the gentlemen licensed are satisfied that the examination was searching and thorough, and whilst some of them may have failed to achieve the highest standard for which they aimed, still they are conscious that the Board did .its duty rigidly, justly and liberally, in consonance with the demands of medical science and the welfare of society.

It is due to the members of the Board from Milledgeville, to say that all who were cognizant of the proceedings of the meeting unite in extolling the profundity, clearness and scope of medical and scientific knowledge in the various departments of the healing art displayed by them.

They have again given additional proof of their high professional standing, if it were at all necessary to prove what all educated physicians throughout the State already know. P.

*Dr. C. H. F., writes us as follows:* "We hope you will urge your readers to advise their students to attend first-class Colleges only, etc."

We agree with our respected correspondent in the belief that students of medicine should attend only Colleges wherein the dignity of the medical profession is conserved by the highest possible culture, and where the science and practice of medicine is taught by the most competent Professors, at the same time it is but just to observe that many believe the existence of mediocre Colleges, presided over by a second-rate faculty, is in a manner necessary in order to accommodate the host of common aspirants to medical honors, who in spite of the dictum of nature and the logic of common sense deem themselves "called" to grasp the scalpel and the pill-box, and relieve the world of all the ills that man is heir to. This is the doctrine of necessary evil, and, by the way, offers a fine and extensive field for discussion for inquiring minds—to such discussion, in a brief and legitimate way, the columns of our Journal are always open. Our desire is to serve the cause of science and elevate the profession. We shall offer our advertising columns to none but those we have reason to believe are worthy of the patronage of the profession. P.

*To Dr. F.*—If the individual alluded to by you, is really as you say, "crazy" and a "liar" constitutionally, we can not give our consent to ventilate said individual's character. It is evident that he is too far gone for any effort of ours to redeem him, no matter how generously or humanely we might undertake his cure. P.

DIED—On the 8th of July, 1874, MRS. V. A. SWEATT, wife of DR. R. P. SWEATT, of Waxahachie, Texas.

We extend to our afflicted professional brother our sincerest condolence in his great grief, and trust that the tender mercy of an all-wise Providence will be abundantly bestowed upon him.

---

### ACKNOWLEGEMENTS.

FOR 1873.

| | |
|---|---|
| Dr E L Crump, Ga | $2 50 |
| Dr M Giesey, Oregon | 2 50 |
| Dr J A Warsow, Miss | 2 50 |
| Dr J W Rickman, Ala | 2 50 |
| Dr W W Mahan, Ark | 2 50 |
| Dr G D Care, Ga | 2 50 |

FOR 1874.

| | |
|---|---|
| Dr John Dickey, Georgia | $2 50 |
| Dr A A Carruth, La | 2 50 |
| Dr Thomas S Jones, La | 2 50 |
| Dr J W Jones, La | 2 50 |
| Dr T J Fentress, Va | 2 50 |
| Dr J H P Stephenson, Miss | 2 50 |
| Dr Robert W Lovett, Ga | 2 50 |
| Dr J B Hughes, N. C | 2 50 |
| Dr M Giesey, Oregon | 2 50 |
| Dr W G Chew, Miss | 2 50 |
| Dr E H Crawford, La | 2 50 |
| Dr W J Luster, Miss | 2 50 |
| Dr A H Randle, Ga | 2 50 |
| Dr P E L Jennings, Ga | 2 50 |
| Dr L C Wisdom, Ga | 2 50 |
| Dr J T Smith, N. C | 2 50 |
| Dr George H Coke, N. C | 2 50 |
| Dr David Cox, N. C | 2 50 |
| Dr E A Morrison, Va | 2 50 |
| Dr L C Samples, Ga | 2 50 |
| Dr J D Harrell, Ala | 2 50 |
| Dr W F Gresham, Ga | 2 50 |
| Dr H T Bahnson, N. C | 2 50 |
| Dr T E Morris, Miss | 2 50 |
| Dr J C Denson, Miss | 2 50 |
| Dr J B Bailey, Miss | 2 50 |
| Dr E M Vasser, Ala | 2 50 |
| Dr William Giles, Miss | 2 50 |
| Dr M C Martin, Ala | 2 50 |
| Dr W P Anderson, Ga | 2 50 |
| Dr J C Clements, Ga | 2 50 |
| Drs Wilson & Webb, Ark | 1 60 |

FOR 1874.

| | |
|---|---|
| Dr S E Winnemore, Ala | $2 50 |
| Dr J R Muse, Tenn | 2 50 |
| Dr A Atkinson, Ga | 2 50 |
| Dr A J Logan, Ga | 2 50 |
| Dr M R Harrison, Miss | 2 50 |
| Dr Wm Gould, New York | 2 50 |
| Dr R L Powell, Va | 2 50 |
| Dr J C McIntosh, Tenn | 2 50 |
| Dr W J Harris, Miss | 2 50 |
| Dr R P Spencer, Va | 2 50 |
| Dr C M Catchings, Miss | 2 50 |
| Dr John Hockenhull, Ga | 2 50 |
| Dr J D Dabney, Miss | 2 50 |
| Dr Thomas L Settle, Va | 2 50 |
| Dr Hugh R Green, Va | 2 50 |
| Dr John Q Winfield, Va | 2 50 |
| Dr J R Allen, Tenn | 2 50 |
| Dr W M Garrard, Ill | 2 50 |
| Dr S W Palmer, Ga | 2 50 |
| Drs Sweatt & Kerr, Texas | 2 50 |
| Dr James T Alford, Miss | 2 50 |
| Dr J B Smith, Ohio | 2 50 |
| Dr W W Mahon, Ark | 2 50 |
| Dr Bennet E Clark, La | 2 50 |
| Dr Alx. J Kalb, La | 2 50 |
| Dr J W Hill, S. C | 2 50 |
| Dr R V Cowan, N. C | 2 50 |
| Dr Thomas J Jones, Ga | 2 50 |
| Dr T H Parham, Va | 1 50 |
| Dr E P Booth, Texas | 2 50 |
| Dr John A Frame, Ohio | 2 50 |
| Dr W H Crawford, Ala | 1 25 |
| Dr G W Booth, Miss | 2 50 |
| Dr R E Nelson, Va | 2 50 |
| Dr Thomas Gibson, Ga | 2 50 |
| Dr V H Talifero, Ga | 2 50 |
| Dr A B Calhoun, Ga | 2 50 |
| Dr George D Case, Ga | 2 50 |
| Dr A A Lyon, Miss | 2 50 |
| Dr J H Hatch, Va | 2 50 |

The above list comprises the names of all subscribers who have paid since the last published list. If any subscriber has forwarded money, and whose name fails to appear in the list, he will please inform us of the fact. Several postal orders are lying in the Post Office, awaiting the arrival of letters of the parties forwarding them. These parties will be informed by letter so as to enable them to send the duplicates.

### ASSOCIATE EDITORS:

**GEORGIA.**

James A. Long, M.D.
W. L. M. Harris, M.D.
H. L Battle, M.D.
W. C. Musgrove, M D
L. B. Bouchelle, M.D.
Thomas Burdell, M.D.
E. H. W. Hunter, M.D.
V. S Hitt, M.D.
J. E. Pope, M.D.
A. H. Brantley, M.D.
J. J. Knott, M.D.
J. C. Wisdom, M.D.
W. W. Leake, M.D.
S. G. WHITE, M.D.
W. H. HALL, M.D.
G. D. CASE, M.D.

**KENTUCKY.**

W. S. Taylor, M.D.
B. W. Stone, M.D.
Charles Kearns, M.D.

**TEXAS.**

S. M. Welch, M.D.
T. J. Heard, M.D.
W. A. Heard, M.D.

**TENNESSEE.**

J. D. Tucker, M.D.

**ALABAMA.**

J. C. Knox, M.D.
Geo. F. Taylor, M.D.
J. B. Barnett, M.D.
S. M. Hogan, M.D.
D. S. Hopping, M.D.
J. T. Searcy, M.D.
S. F. Hill, M.D.

**NORTH CAROLINA.**

J. B. Hughes, M.D.
S. S. Satchwell, M.D.
A. B. Pierce, M.D.
H. Kelly, M.D.
Geo. A. Foote, M.D.
E. A. Anderson, M.D.
H. T. Bahnson, M.D.
Willis Alston, M.D.
Thos. F. Wood, M.D.
N. J. Pittman, M.D.
John W. Booth, M.D.

**SOUTH CAROLINA.**

E. T. McSwain, M.D.
G. M. Rivers, M.D.

**OHIO.**

J. Q. A. Hudson, M.D.
C. H. Tidd, M.D.

**VIRGINIA.**

John H. Claiborne, M.D.
F. Horner, Jr., M.D.
R. J. Preston, M.D.
J. E. Lockridge, M.D.
J. S. Wharton, M.D.
Wm. O. Owen, M.D.
Sam'l P. Ripley, M.D.
Benj. Blackford, M.D.
E. A. Drewry, M.D.
Rob B. Tunstall, M.D.
W. F. Barr, M.D.
L. B. Anderson, M.D.
J. M. Lazzell, M.D.

**MISSISSIPPI.**

C. B. Gallaway, M.D.
Edward Lea, M.D.
S. V. D. Hill, M.D.
E. T. Henry, M.D.
T. P. Lockwood, M.D.
Robert Kells, M.D.

**ARKANSAS.**

A. D. Hodge, M.D.
J. K. Hodge, M.D.
A. W. Hobson, M.D.

**NEW JERSEY.**

J. B. MATTISON, M.D.

### CORRESPONDING EDITORS:

Ely Van DeWarker, M. D., New York.
C. C. F. Gay, M.D., New York, *Surgeon of Buffalo General Hospital.*
M. H. Henry, M.D., New York, *Surgeon to N. Y. Dispensary, Dep't of Venerial and Skin Diseases.*
Benjamin Howard, M.D., New York.
James Tyson, M.D., Philadelphia, Pa.
W. W. Keen, M.D. Philadelphia.
Robert Robson, M D., Indiana.
A. Patton, M.D., Indiana.
John H. Weir, M.D., Illinois.
Thomas J. Gallaher, M. D, Pennsylvania.
S. C. Busey, M.D., Washington, D. C.
Wm. R. Whitehead, M.D., Colorado Territory.

☞ The RECORD will be mailed between the 20th and 25th of ea[illegible] month.

